# Stress among Portuguese Medical Students: A National Cross-Sectional Study

**DOI:** 10.1155/2020/6183757

**Published:** 2020-09-03

**Authors:** Maria João Oura, Ana Raquel Moreira, Paulo Santos

**Affiliations:** ^1^Department of Medicine of Community, Information and Health Decision Sciences (MEDCIDS), Faculty of Medicine, University of Porto, Porto, Portugal; ^2^Center for Health Technology and Services Research (CINTESIS), Faculty of Medicine, University of Porto, Porto, Portugal

## Abstract

**Introduction:**

The medical course is extremely stimulating but also demanding, and it can interfere with students' mental health. Stress leads to lower life quality, academic performance, and ultimately to a lower quality of patient care delivered.

**Objective:**

To analyse stress levels of sixth-year medical students who attend Portuguese colleges. *Methodology*. This observational cross-sectional study involved Portuguese medical students attending the sixth year of all Portuguese faculties. We applied an online self-response questionnaire, including the 10 items Perceived Stress Scale (PSS) to assess stress levels and sociodemographic variables. Logistic regression was used to estimate the weight of the studied determinants on stress levels.

**Results:**

A total of 501 participants were included for analysis (69.5% females), with a median age of 24 years old. We found significant levels of stress in 49.9% (95% CI: 45.5–54.3%), with 20.8% of total students presenting extremely high levels, irrespective of age, gender, and faculty. Stress was higher when students presented bad sleeping and eating habits, lack of ability to manage time, dissatisfaction with social life and academic experience, and low family support. Also, these students are more worried about their future and present a higher degree of concern about their graduation test performance.

**Conclusion:**

This study found high stress levels among Portuguese medical students, associated with social determinants and the intrinsic complexity of the course. This is worrying, and it elucidates the importance of coping strategies to make students deal with stress and be healthier, currently and in the future.

## 1. Introduction

“Stress” is a widely used word in everyday life, but its meaning varies according to the circumstances. Over the years, several conceptual models have been proposed. Hans Selye (1930) described the general adaptation syndrome [1]. He states that stress is the nonspecific response of the body to sensory/psychological stimuli. Selye differentiated two types of stress: the distress (negative stress) and the eustress (positive stress), that are physiologically experienced in a similar way. Currently, however, we know that the cause of stress is not in the event itself, as Selye claimed, but in the physiological response to it. Stress effects become evident not only if the situation is perceived as threatening but when the resources to cope with it are insufficient [2].

There are several strategies to handle stress when it takes over. Coping refers to cognitive, emotional, and/or behavioral efforts to tolerate or reduce a troubled person-environment relationship [3]. Lazarus and Folkman suggested two different perspectives as they focus on the problem or on the emotions. Emotion-focused coping aims to reduce the negative emotions associated with stress and the emotional reaction to the stressors, including measures such as drug therapy, meditation, drinking, reframing, and positive thinking. On the other hand, problem-focused coping attempts to remove or reduce the cause of the stressor. This includes identifying the problem, evaluating possible solutions and choosing one of them [4].

Stress is a natural feeling and an inevitable part of life. It emerges from the combination of a triggering event with the lack of ability to cope with it. Stressors may be linked to work, financial struggles, conflicts in relationships, or major life events. Even though youngsters cope with concerns and anxiety that surround them through optimistic attitudes and a relative positive perception of their future, stress is still common among them. University is an astonishing and challenging experience, both personally and academically. However, it is a period that can exacerbate emotional vulnerabilities. College admission causes significant changes in the students' daily life: entering a new environment, leaving parents' home and accustoming to academic demands may condition stress [5], affecting their physical and emotional welfare and quality of life and leading to lower academic success. They also face concentration difficulties, memory problems, behavior changes, constant worry, self-defeating thoughts, and social withdrawal.

Several studies have demonstrated that medical students suffer more from depression and other mental issues than the general population [6–8], and it may extend even after graduation, into their high emotional demand professional life [9]. This psychological morbidity appears to arise from training process, as medical students show better mental health at admission than age-matched college graduates [10]. This implies the need to provide students better mental health support since medical school to prepare them to a deep emotional medical career.

Medical students may present certain common personality traits. They are generally more perfectionistic, more obsessive-compulsive, and have higher expectations regarding themselves [11], which might contribute to the higher prevalence of mental disorders among them. Nevertheless, it is also these traits that enable them to overcome the adversities throughout those years, and the driving force that helps them to attain their goals.

In Portugal, the medicine course lasts six years, and it requires students to have remarkable dedication, strong emotional capacities, and good time management skills. After graduation, a global test determines the rating for access to future specialty: *Prova Nacional de Acesso* (PNA). The decisive character of this exam to the near future of students provokes a lot of concern during 6^th^ year corresponding to the need to study and prepare the evaluation. Recently, a new model for the exam was introduced, closer to clinical skills, but significantly different from the previous model. Despite a commonly considered positive change, it may be perceived as frightening and stressful.

This study aims to analyse stress levels of medical students attending sixth year of Portuguese Universities, to better understand this problem and draw possible prevention and support measures.

## 2. Materials and Methods

We conducted an observational cross-sectional study in the 6^th^ year grade medical students of Portuguese Universities, by survey.

The total number of students attending the 6th year comprises approximately 1200 individuals, all of them eligible for participation. In Portugal, there are currently eight medical schools. Two of them in Porto (Faculdade de Medicina da Universidade do Porto, FMUP, and Instituto de Ciências Biomédicas Abel Salazar, ICBAS), two in Lisbon (Faculdade de Medicina da Universidade de Lisboa, FML, and NOVA Medical School Lisboa), one in Braga (Escola de Medicina da Universidade do Minho, UM), one in Coimbra (Faculdade de Medicina da Universidade de Coimbra, FMUC), one in Covilhã (Faculdade de Ciências da Saúde da Universidade da Beira Interior, UBI), and one in Faro (Universidade do Algarve, UAlg). Although there are some differences among them, all curricula are quite similar, granting the integrated master grade in Medicine at the end of 6 years, with the exception of UAlg, which targets complementary clinical learning to previous graduate students.

The data collection occurred from September 2019 to the end of January 2020. All 1200 eligible students were invited to participate in the survey on four different occasions through the institutional e-mail system. We also contacted colleges' academic administrations and students' associations in order for them to share our online questionnaire with the respective students. The sample size considering a margin of error of 3.5% for a confidence interval of 95%, for an unknown expectant result, was established in 475 participants.

We measured stress using the Perceived Stress Scale (PSS) developed by Sheldon Cohen and colleagues [2], translated and validated to Portuguese by Pais Ribeiro and Marques [12]. This version has 10 items pointing how often participants have found their lives unpredictable, uncontrollable, and overloaded in the last month, classified by a Likert scale from 0 (never) to 4 (very often). There is a reverse scoring for questions 4, 5, 7, and 8. The final score ranges from 0 to 40, and the higher the score, the greater the stress. It has an internal consistency (Cronbach's *α*) of 0.87 and an item total correlation ranging from 0.32 to 0.82, with the majority above 0.60. In the Portuguese validation, the author explains that by establishing an equivalence between the total score and the respective percentiles, and it was assumed that a score above the 80th percentile was related to a possible pathological state, based on the results obtained in the group of participants with diagnosed anxiety disorders. Therefore, its dichotomic categorization classifies stress as pathological when the score is equal or above 22 in the females and 20 in the males [12]. We also used a polynomial categorization of low stress levels when PSS score ranged from 0 to 13, moderate when it varied from 14 to 26, and high when the score obtained was equal or higher than 27 [13].

We also evaluated social and demographic variables: age, gender, college, enough sleeping hours, eating habits, spirituality, ability to manage time, social life satisfaction, satisfaction with academic experience, personal or family history of mental illness, presence of financial problems, family support, displacement from home, frequency of home visits (if applicable), worries about the future, and the degree of concern about the new exam.

We used descriptive and inferential statistics. Data were encoded and registered in a Microsoft Office Excel 2013® database and analysed using IBM SPSS Statistics®, version 25.0 (IBM Corp., Armonk, NY, USA®). We used descriptive measures to describe our sample. The main outcome “stress” was dichotomized in low or high. Odds ratio for determinants was calculated by logistic regression. A multivariate model, adjusted for age, gender, and faculty of study, was constructed using backward (ward) logistic regression and significant factors in univariate analysis. The significance level was set at 0.05.

The Ethical Committee of Hospital de Sa˜o Joa˜o/Faculty of Medicine of University of Porto assessed and approved the study protocol. We followed the principles of the Helsinki Declaration and the Oviedo Convention about the protection of human rights in the biomedical investigation. The first page of the web-form, before the questionnaire itself, included information for participants, and it also asked for their explicit consent. Participants who refused to consent were automatically excluded from the study.

## 3. Results

We registered 501 valid participants, out of 559 total answers (89.6%). Invalid questionnaires were related to answers from students from other years than the 6^th^ (*n* = 58). As expected by the demography of medical students in Portugal, female respondents were the main group (69.5%), and the mean age was 24 years old (±2.5). The most represented college was FMUP (29.7%), followed by FMUC and FML (19.6% and 13.2%, respectively), which was predictable since these three present the highest attending number of students.  Table 1 shows the demographic characteristics of our sample.

The mean perceived stress score was 20.2 (95% CI: 19.6–20.9). Pathological stress was present in 250 students (49.9%; 95% CI: 45.5–54.3%), using the dichotomic categorization, with no differences between gender, age, or faculty of origin. The polynomial scale showed 89 (17.8%), 308 (61.4%), and 104 (20.8%) students with low, moderate, and high stress levels, respectively.

**Figure 1:**
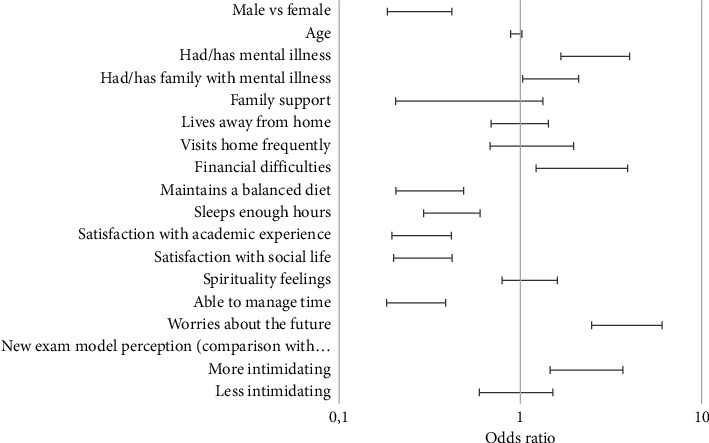
Association of stress determinants with pathological stress (by Perceived Stress Scale score).

In univariate analysis, pathological stress was associated with bad sleeping habits, lack of ability to manage time, and dissatisfaction with social life and academic experience. These students were more worried about their future and presented a worse perception about the graduation test. Besides, 30% of students with stress levels considered pathological had current or past diagnosed mental disease. Stress appeared associated to psychiatric illness.  Figure 1 shows stress determinants and its association with PSS score.

The multivariate analysis using logistic regression adjusted for age, gender, and faculty showed that males were less prone to present stress than females, as those who slept well, ate well, who were satisfied with social life and friends' support, and had a good ability to manage their time (Table 2). Stress was more common among those who presented mental illness, currently or in the past, and those who were worried about their future.

## 4. Discussion

Almost half of the students attending the sixth year of Portuguese medical schools present pathological stress (49.9%), with 20.8% revealing extremely high levels. They are more vulnerable to the development of mental disease, such as anxiety and depression [14]. This is extremely worrying for several reasons. First, youth is supposed to be one of the healthiest and happiest phases in the cycle of life. High stress levels reduce their perceived happiness [15]. Second, it can interfere with study skills, such as concentration and memory capacities, eventually leading to a reduction on academic performance [16]. Finally, there is a risk of persistence throughout life, negatively affecting medical performance and the patients' care and the health care systems.

Higher stress levels are associated with more worries regarding the future and the graduation test performance. This group of students is the second round of the new model of test. They will do the exam in November 2020. There are currently no vacancies available for all candidates, exerting pressure in the performance to achieve the admission for the desired specialty. This is surely a cause of stress, potentially leading to an exacerbation of mental health problems.

Our results also show an association between stress and several social determinants. Difficulties in maintaining healthy eating and sleeping habits, financial problems, lack of ability to manage their time and female gender are associated with pathological stress. In addition, dissatisfaction with social life, academic experience, and the presence of a mental illness make students more prone to high stress levels. All of these factors can not only be the cause but also be the consequence of a stressful situation. Identification and support of students with these risk factors can either prevent the onset and the progression of stress.

These findings are in line with recent studies that also applied PSS to medical students. Samanta and Ghosh (2017), in India, found a mean PSS score of 18.41 (±6.22) in medical students [17], and Tavolacci and Veber (2015), in France, found a mean value of 19.4 (±6.9) in sixth-year students [18]. Rahimi et al. (Canada, 2014) described higher levels of perceived stress in medical students comparing to the general population, matched by age and gender [19]. Even using different methods, Galán (2011), in Spain, found high burnout prevalence in sixth-year students by the Maslach Burnout Inventory [20]. Moreover, several works associate the studied determinants with stress. Hill et al. (2018) described the stressors facing medical students in the millennial generation: medical school workload, performance pressure, financial problems, time constraints, and lack of balance [21]. Several other American [22, 23], European [24, 25], and Asian [26–28] studies have also noted these as stressors among medical students.

In our study, although family support is not associated to pathological stress, it is protective against high stress (OR = 0.211; 95% CI: 0.07–0.64), leading us to hypothesize that family has a relevant role in preventing the extremely high stress in our students. Additionally, feelings of spirituality and displacement from home do not have an impact on stress, unlike some literature showing lack of spirituality [29] and accommodation away from home [30, 31] as severe stressors.

To our knowledge, this is the first nationwide study in Portugal measuring and characterizing perceived stress in the pregraduated medical students and using a highly reliable scale for measuring the perceived stress. Nevertheless, our results must be interpreted with consideration of the limitations of our study. First of all, the cross-sectional design lacks temporality. A longitudinal design would have evaluated temporal relationships. The self-response questionnaire may condition some information bias, in which respondents provide what they believe to be socially acceptable answers rather than their own truth, even using a Likert scale to prevent the tendency for the correct answer. Besides, there was no clinical consultation of stressed subjects. Finally, since the participation in the study was voluntary, there could be a self-selecting bias, in which the students who decided to answer may present different characteristics in comparison to the nonanswering community.

This study emphasizes the high number of medical students presenting pathological stress. In fact, the Portuguese validation of the PSS score showed a mean value of 13.9 in the males and 17.1 in the females, which are both lower than the mean score obtained in this study. However, few students actually seek help [32], which may lead to a persistence of this problem into the medical practice. It is then of extreme importance to identify and implement prevention and management measures since it might prevent students from getting into a vicious cycle of stress and related diseases. A wide range of strategies can be used to cope with stress.

Physical exercise, yoga, or music-related activities may have positive effects on the emotions and the body [33–35]. Therefore, we believe that colleges might reinforce the importance of their extracurricular activities, in an affordable way, in order to augment the students' educational experience outside medical curriculum.

Furthermore, we also suggest the implementation of stress management programs among colleges, as it has demonstrated its efficiency and ability to positively influence students' approach on their well-being. For instance, mindfulness-based stress reduction (MBSR) is a well-established training that has been associated with lower stress levels, anxiety, and depression in health professional students [36]. It highlights the importance of observing situations and thinking in a nonjudgmental, nonreactive, and accepting manner [37]. We propose the implementation of a MBSR program carried by a trained teacher, as it was performed in Bristol Medical School (United Kingdom) [38], or by a previously taught student, following the example of the “Peer2Peer” program of the Medical University of Graz (Austria) [39]. Both of them include daily home practices and weekly sessions that address various topics, such as psychological stress, relaxation techniques, and coping mechanisms. Since alcoholic drinking is a relevant problem between youngers and illicit substances' consumption is increasing in Portugal [40], it is also important to tackle this in future programs. Besides, in order to provide a more accessible and anonymous program, there is also the Mindful Gym course, a DVD-delivered program consisting of a five-week audio-video intervention with two to three-hour sessions per week [41]. This type of programs could also be acquired by colleges and freely distributed to their students. In addition, the development of websites addressing stress management and positive thinking, such as the “Computer Assisted Learning for the Mind” in New Zealand [42] or the “ULifeLine” in the United States of America, is also extremely helpful tools, and similar websites could be easily developed by Portuguese colleges, with audio files and information regarding stress.

Moreover, real-time monitoring through electronic devices is a new way to make students taking sense of their current state, by assessing heart rate, for instance, sending an alert able to remind the need to change attitudes to decrease stress levels [43]. Information should be easily available in accessible sources, with scientific rigor and topicality [44].

We believe that Portuguese colleges have to assume their responsibility when it comes to their students' mental health. There are some optional activities and programs mostly carried out by students' associations, but there is a lack of programs implemented by colleges' board. Whether activities or programs are implemented as part of the core medical school curriculum or as an optional component, it is pivotal to address not only stress management skills but also focus on providing strategies to enhance, for instance, sleep hygiene, academic experience, and time management and to alleviate worries about the future.

There is a wide range of coping and intervention measures useful for medical students. Several studies explain the different methods, but there is a lack of interventional studies that measure their efficacy. In the future, it would be of extreme importance to conduct those studies to assess these strategies' efficacy. Besides, it is imperative to evaluate how these measures should be implemented, whether as a part of a large nationwide program or an intensive course with a few weeks' duration. A study found that UK undergraduate and graduate-entry medical students cope with stress differently, despite having similar profiles of stress symptoms [45]. This needs to be elucidated in future studies as it means that coping mechanisms should be adapted according to the students' situation. Upcoming studies should also assess the temporal relationship between perceived stress among the six years of medical school, as the literature is controversial. A recent study suggests that stress levels do not vary significantly over training years, remaining moderately high [46], but this is not well-established yet. Finally, since saliva, blood, or hair cortisol levels are objective stress measures, it could be interesting to complement stress studies with them [47].

## 5. Conclusion

Medical students' stress levels found in this study are worrying, and it should warn us to the urgent need for the development of prevention and support measures. The medical career is an extremely demanding and never-ending path. These demands already commence in college where students face certain difficulties and challenges that may lead to an exacerbation of stress and, ultimately, to burnout. It is pivotal to strike this problem early instead of allowing it to progress into the medical practice.

## Figures and Tables

**Table 1:** Sociodemographic characteristics of medical students.

Characteristics	*N* (%)
Gender	
Male	153 (30.5%)
Female	348 (69.5%)
Mean age in years (SD)	24.0 (±2.5)
Medical school	
FMUP	149 (29.7%)
ICBAS	57 (11.4%)
EM	37 (7.3%)
UBI	48 (9.6%)
FMUC	98 (19.6%)
FMUL	66 (13.2%)
NOVA	30 (6.0%)
UAlg	16 (3.2%)
Lives away from home	319 (63.7%)
Visits home frequently (in those living away from home)	248 (78.0%)
Sleeps enough hours	248 (49.5%)
Maintains a balanced diet	370 (73.9%)
Able to manage time	283 (56.5%)
Social life satisfaction	216 (43.1%)
Satisfaction with academic experience	208 (41.5%)
Financial problems	57 (11.4%)
Good family support	481 (96.0%)
Feelings of worry about the future	375 (74.9%)
Spirituality feelings	231 (46.1%)
New exam model perception	
Less intimidating	181 (36.1%)
Equally intimidating	121 (24.2%)
More intimidating	199 (39.7%)
Had/has mental illness	17 (23.4%)
Had/has family with mental illness	283 (56.5%)

FMUC, Faculty of Medicine of University of Coimbra; FMUL, Faculty of Medicine of University of Lisbon; FMUP, Faculty of Medicine of University of Porto; ICBAS, Institute of Biomedical Sciences “Abel Salazar”; NOVA, NOVA Medical School; UAlg, University of Algarve; UBI, Faculty of Health Sciences of University of Beira Interior; UM, Faculty of Medicine of University of Minho; SD, standard deviation.

**Table 2:** Factors related with stress in medical students.

Determinants	OR (95%CI)	*p* value
Male vs female	0.194 (0.115–0.328)	<0.001
Sleeps enough hours	0.586 (0.362–0.951)	0.030
Had/has mental illness	2.982 (1.717–5.192)	<0.001
Maintains a balanced diet	0.450 (0.259–0.783)	0.005
Able to manage time	0.391 (0.243–0.627)	<0.001
Social life satisfaction	0.471 (0.282–0.789)	0.004
Satisfaction with academic experience	0.535 (0.320–0.897)	0.018
Worries about the future	3.092 (1.794–5.329)	<0.001

Multivariate analysis of the dichotomic categorization (considering pathological stress levels equal or above 22 in the females and 20 in the males), using logistic regression adjusted for age, gender, and college. (CI: confidence interval; OR: odds ratio; *p* value was set at <0.05).

## Data Availability

The data used to support the findings of this study were supplied by Prof. Paulo Santos under license and so cannot be made freely available. Requests for access to these data should be addressed to Prof. Paulo Santos at Faculty of Medicine of University of Porto, Portugal psantosdr@med.up.pt.
